# 2016 WSES guidelines on acute calculous cholecystitis

**DOI:** 10.1186/s13017-016-0082-5

**Published:** 2016-06-14

**Authors:** L. Ansaloni, M. Pisano, F. Coccolini, A. B. Peitzmann, A. Fingerhut, F. Catena, F. Agresta, A. Allegri, I. Bailey, Z. J. Balogh, C. Bendinelli, W. Biffl, L. Bonavina, G. Borzellino, F. Brunetti, C. C. Burlew, G. Camapanelli, F. C. Campanile, M. Ceresoli, O. Chiara, I. Civil, R. Coimbra, M. De Moya, S. Di Saverio, G. P. Fraga, S. Gupta, J. Kashuk, M. D. Kelly, V. Koka, H. Jeekel, R. Latifi, A. Leppaniemi, R. V. Maier, I. Marzi, F. Moore, D. Piazzalunga, B. Sakakushev, M. Sartelli, T. Scalea, P. F. Stahel, K. Taviloglu, G. Tugnoli, S. Uraneus, G. C. Velmahos, I. Wani, D. G. Weber, P. Viale, M. Sugrue, R. Ivatury, Y. Kluger, K. S. Gurusamy, E. E. Moore

**Affiliations:** General Surgery I, Papa Giovanni XXIII Hospital, Piazza OMS 1, 24127 Bergamo, Italy; Department of Surgery, UPMC, University of Pittsburgh School of Medicine, Pittsburgh, PA USA; Department of Surgical Research, Medical Univeristy of Graz, Graz, Austria; Department of Emergency and Trauma Surgery of the University Hospital of Parma, Parma, Italy; Department of General Surgery, Adria Civil Hospital, Adria (RO), Italy; University Hospital Southampton, Southampton, UK; Department of Traumatology, John Hunter Hospital and University of Newcastle, Newcastle, NSW Australia; Acute Care Surgery, Queen’s Medical Center, School of Medicine of the University of Hawaii, Honolulu, HI USA; Department of Surgery, IRCCS Policlinico San Donato, University of Milan Medical School, Milan, Italy; University of Verona, Verona, Italy; Unit of Digestive, Hepato-Pancreato-Biliary Surgery and Liver Transplantation, Henri Mondor Hospital AP-HP, Université Paris Est–UPEC, Créteil, France; Surgical Intensive Care Unit, Department of Surgery, Denver Health Medical Center, University of Colorado School of Medicine, Denver, USA; General Surgery - Day Surgery Istituto Clinico Sant’Ambrogio, Insubria University, Milan, Italy; Ospedale San Giovanni Decollato – Andosilla, Civita Castellana, Italy; Emergency Department, Trauma Center, Niguarda Hospital, Milan, Italy; Department of Surgery, Auckland City Hospital, Auckland, New Zealand; Division of Trauma, Surgical Critical Care, Burns, and Acute Care Surgery, University of California San Diego Health Sciences, San Diego, CA USA; Harvard University, Cambridge, MA USA; General, Emergency and Trauma Surgery, Maggiore Hospital Trauma Center, Bologna, Italy; Division of Trauma Surgery, University of Campinas, Campinas, SP Brazil; Department of Surgery, Government Medical College, Chandigarh, India; Tel Aviv University Sackler School of Medicine, Assia Medical Group, Tel Aviv, Israel; Acute Surgical Unit, Canberra Hospital, Canberra, ACT Australia; Surgical Department, Mozyr City Hospital, Mozyr, Belarus; Erasmus MC Rotterdam, Rotterdam, Holland Netherlands; University of Arizona, Tucson, AZ USA; Meilahti Hospital, Helsinki, Finland; Department of Surgery, Harborview Medical Center, Seattle, WA USA; Department of Trauma, Hand, and Reconstructive Surgery, University Hospital, Goethe-University Frankfurt, Frankfurt, Germany; Department of Surgery, University of Florida, Gainesville, FL USA; First General Surgery Clinic, University Hospital St. George/Medical University, Plovdiv, Bulgaria; Department of Surgery, Macerata Hospital, Macerata, Italy; Shock Trauma Center, Critical Care Services, University of Maryland School of Medicine, Baltimore, MD USA; Denver Health Medical Center, Denver, CO USA; Taviloglu Proctology Center, Istanbul, Turkey; Department of Surgery, Medical University of Graz, Graz, Austria; Emergency Surgery, and Surgical Critical Care, Massachusetts General Hospital, Boston, MA USA; DHS, Srinagar, Kashmir India; Trauma and General Surgery & The University of Western Australia, Royal Perth Hospital, Perth, Australia; Infectious Disease Unit, Teaching Hospital, S. Orsola-Malpighi Alma Mater Studiorum, University of Bologna, Bologna, Italy; Letterkenny University Hospital & Donegal Clinical Research Academy, Donegal, Ireland; Virginia Commonwealth University, Richmond, VA USA; Division of General Surgery, Rambam Health Care Campus, Haifa, Israel; Royal Free Campus, University College London, London, UK

**Keywords:** Acute calcolous cholecystitis, Diagnosis, Cholecystectomy, Biliary tree stones, Surgical risk, Gallbladder percutaneous drainage, Endoscopic ultrasound, Magnetic resonance, Antibiotic, Abdominal infections

## Abstract

Acute calculus cholecystitis is a very common disease with several area of uncertainty. The World Society of Emergency Surgery developed extensive guidelines in order to cover grey areas. The diagnostic criteria, the antimicrobial therapy, the evaluation of associated common bile duct stones, the identification of “high risk” patients, the surgical timing, the type of surgery, and the alternatives to surgery are discussed. Moreover the algorithm is proposed: as soon as diagnosis is made and after the evaluation of choledocholitiasis risk, laparoscopic cholecystectomy should be offered to all patients exception of those with high risk of morbidity or mortality. These Guidelines must be considered as an adjunctive tool for decision but they are not substitute of the clinical judgement for the individual patient.

## Background

Gallstones are common and present as acute calculus cholecystitis (ACC) in 20 % of patients with symptomatic disease, with wide variation in severity. In developed countries, 10–15 % of the adult population is affected by gallstones. According to the third National Health and Nutrition Examination Survey, 6.3 million men and 14.2 million women aged 20 to 74 in the United States had gallbladder disease [1–5]. In Europe, the Multicenter Italian Study on Cholelithiasis (MICOL) examined nearly 33,000 subjects aged 30 to 69 years in 18 cohorts of 10 Italian regions. The overall incidence of gallstone disease was 18.8 % in women and 9.5 % in men [6]. However, the prevalence of gallstone disease varies significantly between ethnicities. Biliary colic occurs in 1 to 4 % annually [1, 7–9]. ACC occurs in 10 to 20 % of untreated patients [9]. In patients discharged home without operation after ACC, the probability of gallstone related events is 14, 19, and 29 % at 6-weeks, 12 weeks, and at 1 year, respectively. Recurrent symptoms involve biliary colic in 70 % while biliary tract obstruction occurs in 24 % and pancreatitis in 6 % [10]. Despite the relevant frequency of ACC, significant controversies remain regarding the diagnosis and management of ACC. The 2007 and 2013 Tokyo guidelines (TG) attempted to establish objective parameters for the diagnosis of ACC [11, 12]. However debates continue in the diagnostic value of single ultrasound (US) signs, as well as of laboratory tests. With regard to the treatment of ACC, historically, the main controversies were around the timing of surgery. The need for surgery as compared to conservative management has been less investigated, particularly in high surgical risk patients. The other major disagreements include: method and need to diagnose potential associated biliary tree stones during ACC, treatment options, type of surgery, definition and management of high surgical risk patients (with clarification of the role for cholecystostomy).

While the TG have certainly improved the understanding of ACC, some criticisms have followed [13, 14]. Indeed, the references in the TG are outdated for some recommendations; the ACC scoring system has not been validated and it does not distinguish between suspected gallbladder inflammation and systemic signs of ACC. Finally, the conclusions are not clear because all the different therapeutic options are available for the same “cholecystitis severity grade”. For these reasons the World Society of Emergency Surgery (WSES) decided to convene a consensus conference (CC) to investigate these controversies and define its guidelines regarding diagnosis and treatment of ACC.

## Material and methods: consensus conference organizational model

On August 2013 the Scientific Board of the 2^nd^ World Congress of the World Society of Emergency Surgery (WSES), endorsed its president, to organize the CC on ACC in order to develop the WSES Guidelines on this topic. The WSES President appointed four members to a Scientific Secretariat, eight members to an Organization Committee and eight members to a Scientific Committee, choosing them from the expert affiliates of WSES. Eight relevant key questions regarding diagnosis and treatment of ACC (reported in Table 1) were developed to thoroughly analyse and fully cover the topic. Under the supervision of the Scientific Secretariat, a bibliographic search related to these questions was performed by an expert library documentarist (medical library of Papa Giovanni XXIII Hospital of Bergamo, Italy), who provided the results of the electronic search of PubMed and EMBASE through May 2015 without time or language restriction. The key words used for the electronic search are listed in Table 1. An additional manual bibliography search was performed by each of the members of the working groups involved in the analysis of the above mentioned eight questions. Before the CC, a number of statements were developed for each of the main questions, along with the Level of Evidence (LoE) and the Grade of Recommendation (GoR) for each statement. The 2011 Oxford Classification was used to grade the LoE and GoR (available at http://www.cebm.net/explanation-2011-ocebm-levels-evidence/) Provisional statements and their supporting evidence were then submitted for review to all the participating members of the CC and to the WSES board members by email before the CC. Modifications were performed when necessary based on feedback.

**Table 1:** Key questions and key words used to develop the Consensus Conference on Acute Calculous Cholecystitis (ACC)

Key questions	Key words
1) Diagnosis of ACC: investigations.	Acute calculous cholecystitis Diagnosis, Ultrasound, Gallstones disease diagnosis.
2) Treatment of ACC: best options.	Gallstones Dissolution, No-surgery gallstones, Extra-corporeal shock wave lithotripsy, Acute calculous cholecystitis, Gallstone disease, Management Gallstones, Endoscopy, Gallstone removal, Observation gallstones.
3) Antibiotic therapy for ACC.	Antibiotics,Acute calculous cholecystitis, Gallstone disease, Management Gallstones.
4) Patient selection for surgery: risk stratification i.e. definition of high risk patients	Acute calculous cholecystitis, Gallstone disease, Surgical risk score, High risk patient, old patient, PPossum score, Apache score
5) Timing for surgery for ACC	Acute calculous cholecystitis, acute cholecystitis
6) Type of surgery for ACC	Acute calculous cholecystitis, Surgery, Laparoscopy, Laparotomy, Cholecystectomy, Partial cholecystectomy, Subtotal cholecystectomy, Cirrhosis, Pregnancy
7) Associated common bile duct stone: suspicion and diagnosis at the presentation	common bile duct stone; choledocholthiasis; endoscopic ultrasound, MRCP, ERCP,
8) Alternative treatments for high risk patients	Acute calculous cholecystitis, Surgery, Gallbladder Drainage, Percutaneous gallbladder drainage, Cholecystostomy, High Risk Patient

**Fig. 1:**
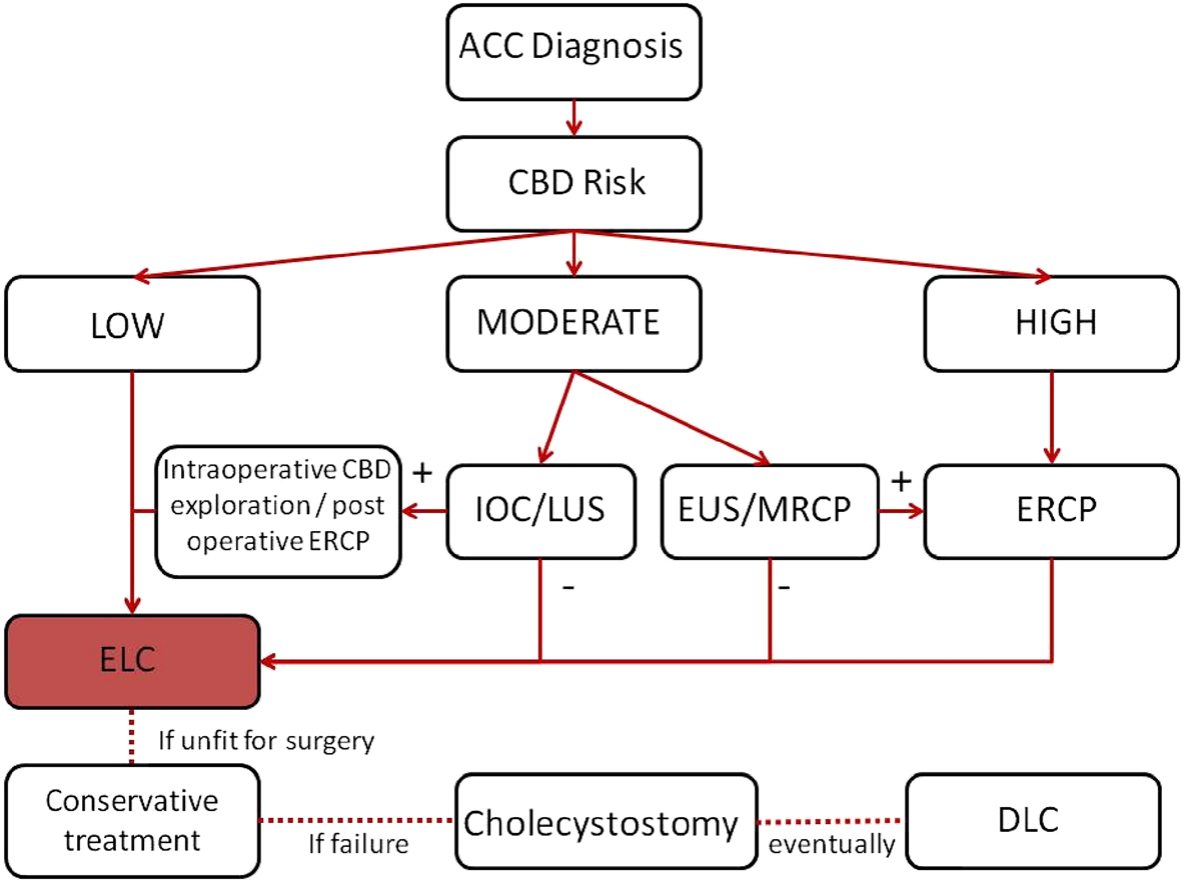
Comprehensive algorithm for the treatment of Acute Calculous Cholecystitis. ACC: acute calculous cholecystitis; CBD: common bile duct; DLC: delayed laparoscopic cholecystectomy; ELC: early laparoscopic cholecystectomy; ERCP endoscopic retrograde cholangiopancreateography; EUS: endoscopic ultrasound; IOC: intraoperative cholangiography; LUS: laparoscopic ultrasound; MRCP magnetic resonance cholangiopancreatography

The CC on ACC was held in Jerusalem, Israel, on July 6th, 2015 during the 3^rd^ World Congress of the WSES. During the first part of the CC, a member of each group presented each of the statements along with LoE, GoR, and the literature supporting each statement. Each statement was then voted upon by the audience in terms of “agree” or “not agree” using an electronic voting system. The percentage of agreement was recorded immediately; in case of disagreement greater than 30 %, the statement was modified after discussion. Furthermore, comments for each statement were collected; the results of vote are available in Appendix 1. Before the second part of the CC, the president and representatives from the Organization Committee, Scientific Committee and Scientific Secretariat modified the statements according to the findings of the first session of the CC. The revised statements were then presented again to the audience. During the CC, a comprehensive algorithm for the treatment of ACC was developed based on the results of the first session of the CC and voted upon for definitive approval (Fig. 1). Simple statements along with their LoE and GoR are available in Appendix 2. Meanwhile all statements are reported in the following Results section, subdivided by each of the eight questions, with the relative discussion and supportive evidence.

These Guidelines must be considered as an adjunctive tool for decision but they are not substitute of the clinical judgement for the individual patient.

## Results

### Diagnosis: investigations

Although ACC is a common disease encountered in the Emergency Department, its diagnosis remains a major challenge. Different diagnostic criteria have been reported in the literature as indicated in the development of the TG [12]. Evidence of an inflamed gallbladder containing stones is the cornerstone for an appropriate diagnosis. The diagnosis of ACC is based on clinical findings, laboratory data, and imaging studies.

#### Statement 1.1 There is no single clinical or laboratory finding with sufficient diagnostic accuracy to establish or exclude acute cholecystitis (LoE 2 GoR B). Combination of detailed history, complete clinical examination, and laboratory tests may strongly support the diagnosis of ACC (LoE 4 GoR C)

A systematic review and meta-analysis of the role of different clinical signs and bedside tests in the diagnosis of ACC included 17 studies in which quantitative assessment of diagnostic values of clinical tests were reported [15]. Twelve variables related to history and clinical examination, 5 variables related to basic laboratory tests, and one variable which was a combination of a clinical sign and a laboratory test were tested in a cohort of patients with abdominal pain or suspected acute cholecystitis. Results showed that with the exception of Murphy’s sign, none of the summary positive likelihood ratios (LR) of the clinical test was higher than 1.6 and none of the summary negative LR was less than 0.4. Murphy’s sign had a positive LR of 2.8 (CI 95 % 0.8 to 8.6) and a negative LR of 0.5 (CI 95 % 0.2 to 1) but the 95 % CI included the value 1. Although the study was classified as one of high quality according to the Oxford classification, it presents some limitations. The study did not report the proportion of patients with abdominal pain and the proportion of patients with suspected acute cholecystitis. Although LR is robust to assess the prevalence, the inclusion of patients with abdominal pain together with patients having suspicion of acute cholecystitis, may be a source of heterogeneity since different pre-test probabilities may be associated with each, modifying the LRs values as a result. Furthermore, reference standards for the definitive diagnosis of acute cholecystitis varied in different studies; this might introduce further bias in the results due to inadequate reference standards. Finally, both ACC and acute acalculous cholecystitis had been included as target condition in this review; the results may have been different if ACC alone had been included as the target condition. In a different prospective diagnostic study, findings from history, clinical examination, and laboratory tests were evaluated in a large cohort of patients complaining abdominal pain [16]. The diagnostic accuracy of a total of 22 variables from the history or clinical symptoms, 15 signs from clinical examinations, and two laboratory tests were evaluated with a reported positive LR of 25.7 and a negative LR of 0.24. The diagnosis was based on the combination of clinical tests without providing details on how such clinical tests had been combined. The study may have a lower strength of evidence, but it refers to a large prospective study including more than 1300 patients.

#### Statement 1.2 Abdominal ultrasound (AUS) is the preferred initial imaging technique for patients who are clinically suspected to have ACC because of its lower cost, better availability, lack of invasiveness, and high accuracy for gallbladder stones(LoE 2 GoR B)

Widespread availability, lack of invasiveness, lack of exposure to ionizing radiation, and a short period of examination are the characteristics that make AUS the first choice imaging investigation for the diagnosis of ACC [17]. To reach the diagnosis of ACC, two conditions must be satisfied: the presence of gallbladder stones and presence of inflammatory changes in the gallbladder wall. There is no doubt that AUS is the best available investigation for the first condition. A meta-analysis by Shea strongly supports this statement. Pooled sensitivity and specificity of AUS in the diagnosis of gallstones were 84 % (95 % CI: 84–92 %) and 99 % (95 % CI: 99–100 %) respectively based on diagnostic accuracy data reported in three studies [18].

#### Statement 1.3 AUS exploration is a fairly reliable investigation method but its sensitivity and specificity for diagnosing ACC is relatively low according to the adopted AUS criteria (LoE 3 GoRC)

Diagnostic performance of AUS in the diagnosis of inflammation of the gallbladder is not as good as its performance in the diagnosis of gallstones, as indicated in a recent meta-analysis [17]. The meta-analysis was based on the results of 26 studies including a total of 2847 patients. The sensitivity in individual studies ranged from 50 to 100 % and specificity from 33 to 100 %; indicating some heterogeneity in the diagnostic performance of AUS. Summary sensitivity was 81 % (95 % CI: 75 to 87 %) and summary specificity was 83 % (95 % CI: 74 to 89 %). However strong heterogeneity was indicated by the inconsistency index, which was reported to be 80 % for sensitivity and 89 % for sensitivity. The review authors have also highlighted that 14 different definitions of positive AUS had been reported in 26 studies; the heterogeneity exploration was however reported to be inconclusive. The quality of studies was not reported to allow a firm conclusion. Two cross-sectional diagnostic accuracy studies of high quality according to the Oxford classification have been published [19, 20]. The criteria for patient selection, diagnostic criteria, reference method, and timing from diagnosis to reference method were sound and well described similarly in both studies. In the study by Hwang et al. [19] which included 107 patients, a sensitivity of 54 % (95 % CI: not reported) and a specificity of 81 % (95 % CI: not reported) were reported by using the combination of sonographic Murphy sign, gallbladder wall thickening greater than 3 mm, peri-cholecystitc fluid collection as major criteria and hepatic biliary dilation and gallbladder hydrops as minor criteria. In the study by Borzellino et al [20] which included 186 patients, diagnostic criteria were assessed using a multivariate analysis. Following the multivariate analysis, distension of the gallbladder, wall oedema, and peri-cholecystic fluid collection were adopted as the criteria for the presence of ACC. The presence of at least one of these three criteria on AUS resulted in a sensitivity of 83.7 % (95 % CI: 75.1 to 89.7 %) and specificity of 47.7 % (95 % CI: 37.6 to 58 %). It appears therefore that AUS may be of limited utility to diagnose or exclude the diagnosis of acute cholecystitis according to the used ultrasound criteria.

#### Statement 1.4 Evidence on the diagnostic accuracy of computed tomography (CT) is scarce. While diagnostic accuracy of magnetic resonance imaging (MRI) might be comparable to that of AUS, insufficient data are available to support it. Hepatobiliary iminodiacetic acid scan (HIDA scan) has the highest sensitivity and specificity for acute cholecystitis, although its scarce availability, long time required to perform the test, and exposure to ionizing radiation limit its use (LoE 2 GoRB)

Because of the poor diagnostic performance of AUS in the diagnosis of ACC, diagnostic accuracy of other imaging modalities must be assessed. A meta-analysis by Kieiwiet et al included studies on CT, MRI, and HIDA in addition to those on AUS [17]. Data on diagnostic accuracy of CT is limited. Kieiwiet et al identified only one study including 49 patients. CT findings of acute cholecystitis included gallbladder distension (41 %), gallbladder wall thickening (59 %), peri-cholecystic fat density (52 %), peri-cholecystic fluid collection (31 %), sub-serosal oedema (31 %) and high gallbladder bile attenuation (24 %) [21]. Thus, there is no single CT feature which is useful in the diagnosis of ACC. Furthermore, the ionizing radiation to which patients are exposed is an issue. CT is therefore usually indicated when sonography is non-diagnostic or patients have confusing signs and symptoms [22]. Kieiwiet et al included three studies on MRI including a total of 131 patients [17]. Summary sensitivity was 85 % (95 % CI: 66 to 95 %) and specificity was 81 % (95 % CI: 69 to 90 %). There was substantial heterogeneity for sensitivity (I^2^ = 65 %) and no heterogeneity for specificity (I^2^ = 0 %). In a head-to-head comparison, diagnostic accuracy of MRI was comparable with that of AUS. The comparison was however based on two studies including only 59 patients; therefore, the strength of evidence is low. Kieiwiet et al included 40 studies with a total of 4090 patients undergoing HIDA scan. Summary sensitivity was 96 % (95 % CI: 94 to 97 %) and specificity 90 % (95 % CI: 86 to 93 %) with no statistically significant heterogeneity for sensitivity (I^2^ = 18 %) but a significant heterogeneity for specificity (I^2^ = 76). In a head-to-head comparison of HIDA with AUS based on 11 studies including a total of 1199 patients, HIDA proved to have better diagnostic accuracy than AUS. The summary sensitivity of HIDA versus AUS was 94 % (95 % CI: 90 to 97 %) and 80 % (95 % CI: 71 to 87 %) respectively with a *P* value < 0.001. The summary specificity of HIDA versus AUS was 89 % (95 % CI: 84 to 92 %) and 75 % (95 % CI: 67 to 82 %) respectively with *P* value < 0.001. As reported in the literature [23] and highlighted by Kieiwiet et al [17], limitation of the information about the biliary tract, the lack of availability of HIDA, and an examination time of several hours strongly shrink the use of HIDA in clinical practice.

#### Statement 1.5 Combining clinical, laboratory and imaging investigations is recommended, although the best combination is not yet known (LoE 4 GoRC)

Combining clinical and AUS findings may improve the diagnostic accuracy; however, studies that report results related to some clinical and imaging combination are few. Hwang et al. [19] reported a 74 % sensitivity and 62 % specificity by combining positive Murphy sign, elevated neutrophil count, and positive AUS. It is interesting to note that within this study, the sensitivity of elevated neutrophil count alone was 79 %; therefore higher than the 74 % sensitivity of combined clinical, laboratory test, and AUS signs. Furthermore, specificity of AUS alone was 81 % which was higher than 62 % reported when combined clinical, laboratory, and AUS findings were analysed.

Another study reported 97 % sensitivity and 76 % specificity by combining C-reactive protein (CRP) and AUS. However, based on the inclusion criteria, generalisability of findings may be an issue in applying the findings to routine clinical practice [24].

The study of Yokoe et al evaluated the Tokyo guidelines criteria and found a sensitivity of 91.2 % and a specificity of 96.9 % of these guidelines in the diagnosis of ACC [12]. Different clinical, laboratory, and imaging findings are combined in the Tokyo guidelines, giving a larger probability to reach the diagnosis. However, the different combinations were not defined in this report. As previously stated, generalisability of these findings to routine clinical practice may be problematic because of the inclusion criteria used in this study.

A full clinical examination should be performed and recorded. This should be combined with laboratory tests for inflammation and AUS. In case of uncertainty in AUS imaging but with a clinical suspicion of ACC, there is no definitive evidence on whether to perform a high cost although highly accurate investigation or to treat the patient empirically as if he or she had ACC.

### Treatment: best options

#### Statement 2.1 There is no role for gallstones dissolution, drugs or extra-corporeal shock wave lithotripsy (ESWL) or a combination in the setting of ACC (LoE 2 GoR B)

The opportunity to dissolve gallstones by medication or break them by ESWL, or combination of both, instead of mechanical removal, has never been tested in the setting of ACC. Strict selection is required to obtain satisfactory results from these therapeutic options: less than 5 mm stone, single stone, cholesterol gallstones, functional gallbladder, and integrity of gallbladder wall when applying external wave to the gallbladder [25]. The rate of recurrence after ESWL is 30 to 50 % at 5 years [26]. Ursodeoxycholic acid was ineffective in a large randomized, double-blind, placebo-controlled trial in patients waiting for elective cholecystectomy in the setting of biliary colic [27]. After gallstone disappearance, the persistence of the same pathogenic factors that induced gallstone formation is primarily responsible for their recurrence after non-surgical treatments of gallstones [28].

#### Statement 2.2 Since there are no reports on surgical gallstone removal in the setting of ACC, surgery in the form of cholecystectomy remains the main option (LoE 4 GoR C)

The opportunity to remove the gallstones in a different way than cholecystectomy has never been tested in the acute setting and the report of this technique are very few. In 2013 Yong et al published the results of 316 consecutive laparoscopic gallbladder-preserving cholelithotomy. The simultaneous use of a choledochoscope to assess the gallbladder clearance appears to drastically reduce the rate of recurrence to 15 % compared to 70 % in the early reports of the 1980’s. The required main patient selection criteria is the functioning gallbladder; this condition is not present in ACC [29].

#### Statement 2.3 Surgery is superior to observation of ACC in the clinical outcome and shows some cost-effectiveness advantages due to the gallstone-related complications and to the high rate of readmission and surgery in the observation group (LoE 3 GoR C)

We found only one prospective randomized study comparing observation to surgery after ACC, published in 2011 by Shmidt [30]. The population size was 33 patients assigned to observation versus 31 assigned to surgery. After an average follow up period of 14 years, 33 % (11 patients) in the observation experienced relapse of gallstones disease (8/11: ACC) and all required surgery. After five years the relapse of symptoms was described as negligible. Despite the value of a long follow-up, the study is underpowered as recognized by the authors themselves. Furthermore, of the eligible patients, 41.3 % were excluded for unknown reasons and the randomization methods were not reported either. Clinical Evidence in 2014 rated this study as moderate/low quality [31]. On the basis of the Shmidt study on ACC and a RCT on symptomatic but uncomplicated gallstone disease [32], Brazzelli et al. produced a clinical and cost-effectiveness analysis, comparing surgery to observation, using an UK based economic model. They found that patients randomized to observation experienced a higher rate of gallstone-related complications (14 % versus 2 %) when compared to surgical group; this happened more frequently in patients with ACC than in those with biliary colic only. From the economic point of view, the frequency of surgery in the observational group (with the need for readmission) slightly favoured surgery. The authors concluded with words of caution because the number of patients was small. In addition, not all aspects were analysed (e.g. abdominal pain in the long term follow up in patients underwent surgery, pain medications cost in the observational group patients, number of visit to the General Practitioner in both groups for biliary related symptoms, etc.) [33, 34].

#### Statement 2.4 Antibiotics should be suggested as supportive care; they are effective in treating the first episode of ACC but a high rate of relapse can be expected. Surgery is more effective than antibiotics alone in the treatment of ACC. (LoE 2 GoR C)

Although ACC is an inflammatory process at the beginning, a secondary infection can occur in the case of continuous bile stasis due to cystic duct occlusion by calculus and oedema, which can lead to sepsis. While many clinicians advocate routine administration of antibiotics in all patients diagnosed with acute cholecystitis, others restrict the antibiotics to patients likely to develop sepsis on the basis of clinical, laboratory, and imaging findings [35]. As a consequence, antibiotics constitute the primary therapy in patients undergoing delayed surgery or observation. In a meta-analysis including 9 RCT on early or delayed cholecystectomy, Papi et al. reported that of 503 patients in the delayed group, 9.3 % experienced a primary failure of antibiotics and supportive therapy and almost 15 % who initially responded suffered recurrences. The rate of unplanned surgery was 26.5 % and a total of 23 % had a failure of conservative treatment [36]. Similar results were reported later in the Cochrane review including only laparoscopic cholecystectomy by Gurusamy in 2013. Approximately, 18.3 % of patients had relapse of symptoms during the waiting period when treated by antibiotics and delayed laparoscopic cholecystectomy for ACC [37]. In 2012 de Mestral et al. published a Ontario-Canada population-based analysis between 2004 and 2011. They collected 25,397 patients with ACC. About 41 % of these patients were not operated at the index admission. Gallstone-related events were measured at 6 weeks, 12 weeks and at 1 year. The respective rates were 14, 19 and 29 %. Pancreatitis and common biliary tract obstruction accounted for 30 % of these events. Gallstone-related events were more frequent in patients aged between 18 and 34 years old [10].

#### Statement 2.5 Cholecystectomy is the gold standard for treatment of ACC (LoE 3 GoR C)

#### Statement 2.6 If surgery is not available, medications such as antibiotics and analgesic should be prescribed and the patients should be referred to a surgical center (depending upon the general condition) due to the high rate of gallstone-related events (LoE 5 GoR D)

Non-surgical options (such as gallbladder drainage) can be considered in surgical high risk patients. The role of non-surgical options will be analysed in a different section.

### Antibiotic therapy

Therapy with appropriate antimicrobial agents is an important component in the management of patients with ACC [38, 39]. Antibiotics are always recommended in complicated cholecystitis and in delayed management of uncomplicated cholecystitis.

#### Statement 3.1 Patients with uncomplicated cholecystitis can be treated without post-operative antibiotics when the focus of infection is controlled by cholecystectomy (LoE 1 GoR B)

In a recently published prospective randomised controlled trial [40], a total of 414 patients treated at 17 medical French centres for grade I or II ACC and who received 2 g of amoxicillin plus clavulanic acid three times a day and once at the time of surgery were randomized after surgery to an open-label, non-inferiority, randomized clinical trial between May 2010 and August 2012. Patients were randomized to either no antibiotics after surgery or continuation with the preoperative antibiotic regimen three times daily for 5 days. An imputed intention-to-treat analysis of the 414 patients showed that the postoperative infection rates were 17 % (35/207) in the non-treatment group and 15 % (31/207) in the antibiotic group (absolute difference, 1.93 %; 95 % CI, -8.98 to 5.12 %). In the per-protocol analysis, which involved 338 patients, the corresponding rates were both 13 % (absolute difference, 0.3 %; 95 % CI, -5.0 to 6.3 %). Among patients with mild or ACC who received preoperative and intra-operative antibiotics, lack of postoperative treatment with amoxicillin plus clavulanic acid did not result in a greater incidence of postoperative infections.

#### Statement 3.2 In complicated acute cholecystitis, the antimicrobial regimens depend on presumed pathogens involved and risk factors for major resistance patterns (LoE 3 GoR B)

The principles of empiric antibiotic treatment should be defined according to the most frequently isolated microbes, always taking into consideration the local trend of antibiotic resistance. Organisms most often isolated in biliary infections are the gram-negative aerobes, *Escherichia coli* and *Klebsiella pneumonia* and anaerobes, especially *Bacteroides fragilis* [41, 42]. Pathogenicity of Enterococci in biliary tract infections remains unclear and specific coverage against these microorganisms is not routinely suggested for community-acquired biliary infections [43]. For selected immunosuppressed patients, i.e. those with hepatic transplantation, enterococcal infection should always be presumed and treated [44]. The main antimicrobial resistance is due to extended spectrum beta-lactamase (ESBL) producing *Enterobacteriaceae*. It is found frequently in community acquired infections in patients with co-morbidities requiring frequent exposure to antibiotic treatments [41, 42]. Health care-related infections are commonly caused by more resistant strains. For these infections, complex regimens with broader spectra are recommended as adequate empiric therapy appears to be a crucial factor affecting postoperative complications and mortality rates, especially in critically ill patients [44]. Although there are no clinical or experimental data to support the use of antibiotics with biliary penetration for these patients, the efficacy of antibiotics in the treatment of biliary infections may depend on effective biliary antibiotic concentrations too. However, in patients with obstructed bile ducts, the biliary penetration of antibiotics may be poor and effective biliary concentrations are reached only in a minority of patients [45]. Antibiotics biliary penetration ability (indicated as the ratio of bile to serum concentrations) are listed in Table 2 [46].

**Table 2:** Antibiotics commonly used to treat biliary tract infections and their biliary penetration ability [46]

Good penetration efficiency (ABSCR > =1)	Low penetration efficiency (ABSCR <1)
Piperacillin/tazobactam (4.8)	Ceftriaxone (0.75)
Tigecycline (> 10)	Cefotaxime (0.23)
Amoxicillin/clavulanate (1.1)	Meropenem (0.38)
Ciprofloxacin (> 5)	Ceftazidime (0.18)
Ampicillin/Sulbactam (2.4)	Vancomycin (0.41)
Cefepime (2.04)	Amikacin (0.54)
Levofloxacin (1.6)	Gentamicin (0.30)
Penicillin “G” (>5)	
Imipenem (1.01)	

*ABSCR* Antibiotics Bile/Serum Concentration Ratio

The choice of the antimicrobial regimen may be problematic in the management of critically ill patients with ACC. In patients with severe sepsis or septic shock of abdominal origin, early correct empirical antimicrobial therapy has a significant impact on the outcome [47]. In a prospective observational study involving 180 consecutive patients with secondary generalized peritonitis, Riché et al. [48] demonstrated a significantly higher mortality rate in septic shock than in those without septic shock (35 versus 8 %).

Recent international guidelines for the management of severe sepsis and septic shock (Surviving Sepsis Campaign) [49] recommend broad-spectrum intravenous antibiotics with good penetration into the presumed site of infection within the first hour. In the event of biliary sepsis, drug pharmacokinetics may be altered significantly in patients with severe sepsis and septic shock. Dosage of antibiotics should be reassessed daily, based on both the pathophysiological status of the patient and the pharmacokinetic properties of the employed antibiotics [50].

#### Statement 3.3 The results of microbiological analysis are helpful in designing targeted therapeutic strategies for individual patients to customize antibiotic treatment and ensure adequate antimicrobial coverage in patients with complicated cholecystitis and at high risk for antimicrobial resistance. (LoE 3 GoR C)

Identifying the causative organism(s) is an essential step in the management of ACC, especially in patients at high risk for antimicrobial resistance such as healthcare-associated infections. It has been reported that positive rates of either bile or gallbladder cultures range from 29 to 54 % for acute cholecystitis [51–58]. In Table 3 are reported the antimicrobial regimens suggested for ACC.

**Table 3:** Antimicrobial regimens suggested for acute calculous cholecystitis

Community acquired	Health-care associated
1) Beta-lactam/beta-lactamase inhibitor combinations based regimens AMOXICILLIN/CLAVULANATE (in stable patients) TICARCILLIN/CLAVULANATE (in stable patients) PIPERACILLIN/TAZOBACTAM (in unstable patients) 2) Cephalosporins based regimens CEFTRIAZONE + METRANIDAZOLE (in stable patients) CEFEPIME + METRANIDAZOLE (in stable patients) CEFTAZIDIME + METRANIDAZOLE (in stable patients) CEFOZOPRAM + METRANIDAZOLE (in stable patients) 3) Carbapenem based regimens ERTAPENEM (in stable patients) IMIPENEM/CILASTATIN (only in unstable patients) MEROPENEM (only in unstable patients) DORIPENEM (only in unstable patients) 4) Fluoroquinolone based regimens (In case of allergy to beta-lactams) CIPROFLOXACIN + METRONIDAZOLE (only in stable patients) LEVOFLOXACIN + METRONIDAZOLE (only in stable patients) MOXIFLOXACIN (only in stable patients) 5) Glycylcycline based regimen TIGECYCLINE (in stable patients if risk factors for ESBLs)	TIGECYCLINE + PIPERACILLIN/TAZOBACTAM (in stable patients) IMIPENEM/CILASTATIN +/- TEICOPLANIN (only in unstable patients) MEROPENEM +/- TEICOPLANIN (only in unstable patients) DORIPENEM +/- TEICOPLANIN (only in unstable patients)

### Patient selection for surgery: risk stratification (i.e. definition of high risk patients)

ACC is a heterogeneous condition. The severity of inflammation and its life-threatening potential is strongly determined by the general status of the patient. It could be argued that alternative treatment to early cholecystectomy could be of benefit for patients with reduced functional reserve. Our search reviewed the available literature to identify the parameters to stratify the risk of surgery in this population and verify if there is any available method to select the best course of action in selected high-risk groups.

#### Statement 4.1 Patient’s age above 80 in ACC is a risk factor for worse clinical behaviour, morbidity and mortality. (LoE 3 GoR B)

Several studies identify old age as a perioperative risk factor for cholecystectomy. However, it is not clear if early laparoscopic cholecystectomy is the best treatment option for elderly patients with ACC. In the retrospective cohort study by Kirshtein et al, the age groups above and below 75 showed a significant difference in mortality (4.8 % versus 0.5 %), morbidity (31 % versus 15 %), and average hospital stay (3.9 versus 2.8) [59]. A recent study by Nielsen et al reported that the odds ratio for mortality in ACC patients older than 80 years with low anaesthetic risk (American Score of Anaesthesiologist I-II (ASA) was significantly higher than in the age groups of 65 to 79 and 50 to 64 (30.9 % vs 5.5 % vs 1 %) [60]. According to Girgin et al, patients’ age, Mannheim peritonitis index ≥29, and co-morbidities are significantly related to morbidity, while increased age and low WBC count are significantly related to mortality in gangrenous cholecystitis [61]. In the case series by Lupinacci et al, mortality of patients older than 80 years was 34.2 % in urgent cholecystectomy versus 0 % in both the elective and semi-elective groups. Statistically significant differences were also demonstrated in morbidity and length of hospital stay. However, the study showed a significantly higher incidence of patients with ASA score of III and IV in the urgent cholecystectomy group (76 % versus 25.6 % versus 28.6 %), and a notably lower number (20 % versus 81.3 % versus 82.8 %) of laparoscopic cholecystectomies [62].

Few retrospective cohort studies compare the outcome of early versus delayed cholecystectomy in aged ACC patients. They fail to demonstrate a significant difference in mortality and postoperative complications [63–66]. A study by Cull et al showed that recurrent episodes of pancreatitis, cholecystitis, and cholangitis were significantly less likely after early than delayed cholecystectomy, irrespective of whether delayed cholecystectomy was preceded by percutaneous cholecystostomy [65]. These findings confirmed the results of a recent population-based analysis on a sample of the Medicare Claims Data System. In this analysis, a lack of a definitive surgical treatment at the index admission in an aged population is associated with 38 % gallstone-related readmission rate in two years versus 4.4 % in similar patients who had early cholecystectomy [67].

#### Statement 4.2 The co-existence of diabetes mellitus does not contraindicate urgent surgery but must be re-considered as a part of the overall patient comorbidity (LoE 3 GoR C)

In 1995, Shpitz et al showed a greater incidence of cardiovascular disease and associated bacterobilia in diabetics who underwent urgent cholecystectomy for ACC; however, they did not report a significant difference in the postoperative outcome [68]. A recent analysis of a large ACC cholecystectomy series from the American College of Surgeons National Surgical Quality Improvement Program database demonstrated that diabetes increased the risk of mortality (4.4 % versus 1.4 %, adjusted odds ratio (OR) 1.79 (95 % CI: 1.09 to 2.94), adjusted *P* value = 0.022), cardiovascular events (2.3 versus 0.5 %; OR 2.50 (95 % CI: 1.25 to 4.99); adjusted *P* value = 0.010), and renal failure (2.5 versus 0.3 %; OR 3.91 (95 % CI: 1.82 to 8.40); adjusted *P* value = 0.001) [69]. A second study on the same series showed that delay in surgery in diabetic patients was associated with significantly higher odds of developing surgical site infections and a longer hospital stay. The same findings were not found in the non-diabetic patients of the same series [70], suggesting that a prompt course of action is appropriate in diabetics.

#### Statement 4.3 Currently, there is no evidence of any scores in identifying patient’s risk in surgery for ACC. ASA, POSSUM and APACHE II are correlated to surgical risk in patients with gallbladder perforation, higher accuracy being for APACHE II. However, APACHE II is built to predict morbidity and mortality in the patients admitted to ICU: its use as a preoperative score should be considered as an extension usage from the original concept. (LoE 4 GoR C). Therefore, prospective and multicentre studies to compare different risk factors and scores are necessary

None of the available clinical scores for the evaluation of surgical risk for acute conditions has been validated for ACC. Recently, the Tokyo guidelines attempted to address the heterogeneity of the ACC population with a therapeutic algorithm that includes some elements of risk stratification. They suggest a staging system based upon severity assessment criteria such as degree of local inflammation and patient conditions, without including any of the most commonly adopted risk stratification scores [71]. However, their classification lacks a clinical validation and has not been validated by studies showing an improved outcome after its introduction. In fact, a retrospective series failed to find any significant benefit [13]. In 2006, Yi et al stratified the risk in relation to the ASA score. The study shows a significant difference in morbidity (20 % versus 9.1 %) in patients in ASA III vs ASA I, with no significant difference in the conversion rate, recovery time or hospital postoperative stay [72] The only available comparison of risk assessment scores (ASA, APACHE II and POSSUM) is limited to series of perforated ACC. The study highlights a significant association of the three scores with morbidity and mortality. Both POSSUM and APACHE II were superior to ASA in risk prediction [73]. Finally, we would like to point out that the usefulness of any score is to add but not to trump surgical judgement: in other words not all patient variables (e.g. recent coronary stent or recent pulmonary embolism, etc.) will be included in any score.

### Timing for surgery: what is early cholecystectomy?

Several randomised controlled trials have investigated early laparoscopic cholecystectomy versus delayed laparoscopic cholecystectomy [74–82].

Early and delayed laparoscopic cholecystectomy have been defined differently in different trials. In general, early laparoscopic cholecystectomy has been defined variably as that performed in patients with acute cholecystitis with symptoms less than 72 h or symptoms less than 7 days but within 4 to 6 days of diagnosis. This roughly translates to 10 days from onset of symptoms. The delayed laparoscopic cholecystectomy is defined variably as that performed between 7 days to 45 days and that performed at least 6 weeks after initial diagnosis.

#### Statement 5.1 Early laparoscopic cholecystectomy is preferable to delayed laparoscopic cholecystectomy in patients with ACC as long as it is completed within 10 days of onset of symptoms (LoE 1 GoR A)

Different patients were included in the trial and the definitions of early laparoscopic cholecystectomy used by these trials comparing early laparoscopic cholecystectomy versus delayed laparoscopic cholecystectomy performed within 6 weeks after initial diagnosis were different in various studies. Six trials provided clinical results. Overall, the systematic review and meta-analysis of randomised controlled trials which included clinical data from five of these six trials demonstrated no significant difference in the complication rate or conversion to open cholecystectomy between early and delayed laparoscopic cholecystectomy and a hospital stay which was statistically shorter by 4 days in the early laparoscopic cholecystectomy group compared to the delayed laparoscopic cholecystectomy group [37]. One trial which was not included in the systematic review also showed similar results as the systematic review (i.e. there was no significant difference in the complication rate between early and delayed laparoscopic cholecystectomy and the hospital stay was shorter by 4 days in the early laparoscopic cholecystectomy group compared to the delayed laparoscopic cholecystectomy group) despite including participants with symptoms > 72 h [81].

#### Statement 5.2 Laparoscopic cholecystectomy should not be offered for patients beyond 10 days from the onset of symptoms unless symptoms suggestive of worsening peritonitis or sepsis warrant an emergency surgical intervention. In people with more than 10 days of symptoms, delaying cholecystectomy for 45 days is better than immediate surgery (LoE 2 GoR B)

One trial compared early laparoscopic cholecystectomy versus delayed laparoscopic cholecystectomy performed between 7 days and 45 days after initial diagnosis [83]. In this trial, the duration of symptoms in the participants was not reported. early laparoscopic cholecystectomy was performed within 24 h of admission while delayed laparoscopic cholecystectomy was performed between 7 days and 45 days. This trial demonstrated that the morbidity was higher in the delayed laparoscopic cholecystectomy compared to early laparoscopic cholecystectomy group and the length of hospital stay was 5 days longer in the delayed laparoscopic cholecystectomy group compared to early laparoscopic cholecystectomy group [83]. There was no significant difference in the conversion to open cholecystectomy between the two groups [83].

#### Statement 5.3 Early laparoscopic cholecystectomy should be performed as soon as possible but can be performed up to 10 days of onset of symptoms. (Level 1 Evidence; Grade A recommendation). However, it should be noted that earlier surgery is associated with shorter hospital stay and fewer complications (LoE 2 GoR B)

One randomised controlled trial compared early laparoscopic cholecystectomy as soon as surgical schedule allows with early laparoscopic cholecystectomy after resolution of symptoms but within 5 days of admission [74] in patients with ACC. The duration of symptoms prior to admission was not reported in this trial. There was no statistically difference in the complication rate or conversion to open cholecystectomy between patients who underwent surgery as soon as the scheduling allowed compared to those who underwent surgery after resolution of symptoms but within 5 days of admission [74]. However, the length of hospital stay was shorter in patients who underwent surgery as soon as the scheduling allowed compared to those who underwent surgery after resolution of symptoms but within 5 days of admission [74]. Evidence from a large database review including approximately 95,000 patients with ACC demonstrated that patients who had surgery within 2 days of admission had fewer complications than those who underwent surgery between 2 and 5 days of admission, and those who had surgery between 6 days and 10 days of presentation. There was no significant difference in the groups between conversion to open surgery [84]. Finally, several studies suggest that cholecystectomy performed as soon as possible, especially in the scenario of an Acute Care Surgery Service, is cost-effective [83, 85, 86].

### Type of surgery

#### Statement 6.1 In ACC, a laparoscopic approach should initially be attempted except in case of absolute anaesthesiology contraindications or septic shock (LoE 2 GoR B)

According to Tokyo Guidelines 2013 (TG13), laparoscopic cholecystectomy is now accepted as a safe surgical technique when it is performed by expert surgeons even in the setting of ACC. TG13 described the surgical treatment of ACC according to the degree of severity of the disease. early laparoscopic cholecystectomy is indicated for patients with Grade I (Mild) ACC. early laparoscopic cholecystectomy is indicated also for patients with Grade II (Moderate) ACC in experienced centers, but in the case of severe signs of local inflammation (WBC > 18.000; a palpable tender mass in the right upper quadrant and >72 h from the onset) should be indicated a conservative treatment with gallbladder drainage followed by a delayed cholecystectomy. For patients with severe local complications such as biliary peritonitis, emphysematous cholecystitis, gangrenous cholecystitis and purulent cholecystitis, emergency surgery is conducted (open or laparoscopic) along with the usual supportive measures. For Grade III (Severe) ACC, TG13 suggest gallbladder drainage and delayed cholecystectomy after improvement of general clinical conditions [71]. Some Scientific Societies also support, more strongly than TG13, laparoscopic cholecystectomy in ACC as the first line approach [87–89].

#### Statement 6.2 Laparoscopic cholecystectomy for ACC is safe, feasible, with a low complication rate and associated with shortened hospital stay (LoE 1 GoR A)

Although Borzellino et al. in their meta-analysis suggested that laparoscopy is not indicated for all cases of ACC due to the difficulty of cholecystectomy in patients with severe inflammation [90], several recent case control, randomized clinical trials have compared laparoscopic cholecystectomy to open cholecystectomy in ACC [91–100]. A recently published meta-analysis demonstrated that laparoscopic cholecystectomy in ACC is the preferable approach with lower mortality and morbidity, significantly shorter postoperative hospital stay and reduced rate of pneumonia and wound infections, compared to the open technique. Conversion rate ranged from 8 to 35 % [101].

#### Statement 6.3 Among high-risk patients, in those with Child A and B cirrhosis, advanced age >80, or pregnant women, laparoscopic cholecystectomy for ACC is feasible and safe (LoE 3 GoR C)

Some studies suggested that laparoscopic cholecystectomy should be the first line approach in specific categories of patients such as the elderly or pregnant women [102, 103]. According to meta-analysis published by de Goede et al., elective laparoscopic cholecystectomy in patients with Child A or B cirrhosis is associated with significantly less postoperative complications, shorter duration of hospitalization and shorter time to resume normal diet compared to open technique [104]. According to Lucidi et al. laparoscopic cholecystectomy should be recommended as the first choice approach in cirrhotic patients; however recommendation for laparoscopic cholecystectomy in patients with Child C cirrhosis is not clear [105]. Cirrhosis is a major risk factor for surgery. laparoscopic cholecystectomy in cirrhotic patients is associated with significantly prolonged duration of surgery, increased operative blood loss, conversion rate, hospital stay and overall morbidity and mortality when compared with non-cirrhotic patients [106]. Laparoscopic cholecystectomy-related morbidity in cirrhotic patients is directly related to the Child Pugh score [107, 108]. In patients with advanced cirrhosis and severe portal hypertension, specific technical difficulties may be encountered, due to the presence of a portal cavernoma, the difficulty in exposure of Calot’s triangle and dissection of the gallbladder hilum, the presence of adhesions and neovascularization or the difficulty in controlling bleeding from the liver bed. Subtotal cholecystectomy can avoid many of these difficulties [109]. In conclusion, laparoscopic approach should be the first choice for the cholecystectomy in Child A and B patients. The approach to patients with Child Pugh C no-compensated cirrhosis remains a matter of debate. As a first recommendation, cholecystectomy should be avoided in these patients, unless clearly indicated, such as in ACC not responding to antibiotics [105].

#### Statement 6.4 Laparoscopic or open subtotal cholecystectomy is a valid option for advanced inflammation, gangrenous gallbladder, or any setting of the “difficult gallbladder” where anatomy is difficult to recognize and main bile duct injuries are more likely (LoE 2 GoR A)

A recent systematic review with meta-analysis by Elshaer et al. reported that subtotal cholecystectomy was performed using the laparoscopic (72.9 %), open (19.0 %) and laparoscopic converted to open (8.0 %) techniques. The most common indications were severe cholecystitis (72.1 %), followed by cholelithiasis in liver cirrhosis and portal hypertension (18.2 %) and empyema or perforated gallbladder (6.1 %). They concluded that subtotal cholecystectomy is an important tool in the difficult cholecystectomy and achieves morbidity rates comparable to those reported for total cholecystectomy in simple cases [110]. Alternative surgical strategy is the fundus first approach to reach progressively the infundibulum, cystic duct and artery: also by using this thecnique the risk of lesions must be always kept in mind [111, 112].

#### Statement 6.5 In case of local severe inflammation, adhesions, bleeding in Calot’s triangle or suspected bile duct injury, conversion to open surgery should be strongly considered. (LoE 3 GoR B)

Tang et al. in their systematic review, identified the principal risk factors for conversion during laparoscopic cholecystectomy. Single factors that appear to be important include male gender, extreme old age, morbid obesity, cirrhosis, previous upper abdominal surgery, severe acute and chronic cholecystitis, and emergency laparoscopic cholecystectomy. The combination of patient and disease related risk factors increases the conversion rate [113]. According to Giger et al., extensive inflammation, adhesions and consequent increased oozing can make laparoscopic dissection of Calot’s triangle and recognition of the biliary anatomy hazardous and difficult. Therefore, conversion to open surgery is strongly recommended to secure patient safety in such difficult conditions [114]. An elevated WBC count (>18 × 10(9)/L) and fever > 38 °C are predictive for the development of complications and conversion [115]. Sugrue et al. recently published the proposal of a new scoring system to evaluate the intraoperative difficulty of the cholecystectomy in order to provide objective suggestion for conversion to open technique [116] and results may clarify and standardize the definition of “difficult surgery”. According to Eldar et al. the complication rate in ACC tended to be associated with duration of complaints >48 h, gangrenous cholecystitis, male sex, age >60 years, other associated diseases, larger bile stones and elevated serum bilirubin levels. Generally, laparoscopic cholecystectomy is safe in all forms of ACC, with acceptably low conversion and complication rates, [117] excluding gangrenous cholecystitis where a conversion rate range between 4 to 40 % [87, 117]. In conclusion gangrenous gallbladder, obscure anatomy, bleeding, bile duct injuries, adhesions and previous upper abdominal surgery represent clinical conditions for which conversion to open cholecystectomy should be strongly considered [118].

### Associated common bile duct stone: suspicion and diagnosis at the presentation

Choledocholithiasis, i.e. the presence of common bile duct stones (CBDS), is reported ro occur in10% to 20 % in case series of cholelithiasis, with lower incidence during ACC ranging from 5 to 15 % of the patients [119–122]. Investigation for CBDS require time and can delay the surgical intervention. Due to the relatively low incidence of CBDS during ACC, the issue is to select patients with a high likelihood of CBDS who would benefit from further diagnostic tests and eventually the removal of the stones. An uncommon condition that mimics CBDS is the Mirizzi syndrome which occurs in 1 % of patients with cholelithiasis: preoperative investigation may help in the diagnosis although the vast majority are identified at surgery [123, 124].

#### Statement 7.1 Elevation of liver biochemical enzymes and/or bilirubin levels are not sufficient to identify ACC patients with choledocholithiasis and further diagnostic tests are needed. (LoE 2 GoR B)

Liver biochemical tests historically have a great utility in determining the presence of CBDS. However, the majority of published studies are not in patients with ACC and also include asymptomatic cholelithiasis. Normal liver biochemical tests have a negative predictive value of 97 %, whereas the positive predictive value of any abnormal liver biochemical test result is only 15 % [125]. Positive predictive value of liver function studies is a poor tool for prediction of CBDS, even in non-ACC, with results ranging from 25 to 50 % [119, 126, 127]. In fact, in ACC, liver biochemical tests may be altered due to the acute inflammatory process of the gallbladder and the biliary tree. 15 to 50 % of patients with ACC show elevation in liver enzymes without choledocholithiasis. Song et al demonstrated that 424 of 1178 patients with ACC had increased liver tests (alanine transaminase (ALT), aspartate transaminase (AST) greater than twice normal levels). Of these only 246 (58 %) had choledocholithiasis [128]. Chang et al showed that 51 and 41 % of ACC patients without choledocholithiasis had elevated ALT and AST, respectively. However, increased bilirubin levels with leukocytosis may predict gangrenous cholecystitis [129]. Padda et al demonstrated that approximately 30 % of patients with ACC without choledocholithiasis had abnormal alkaline phosphatase (ALP) and/or bilirubin and 50 % had abnormal ALT. Among patients with ACC and choledocholithiasis, 77 % had abnormal ALP, 60 % abnormal bilirubin and 90 % elevated ALT. By multivariate analysis increased common bile duct size and elevated ALT and ALP were predictors of choledocholithiasis [130]. The diagnostic accuracy increases for cholestasis tests such serum bilirubin with the duration and the severity of obstruction. Specificity of serum bilirubin level for CBDS was 60 % with a cut-off level of 1.7 mg/dL and 75 % with a cut-off level of 4 mg/dL [126]; however, mean level of bilirubin in patients with CBDS is generally lower (1.5 to 1.9 mg/dL) [119, 127]. In a prospective study, Silvestein reported the diagnostic accuracy of serum bilirubin and serum ALP at two cut-offs for each test. Serum bilirubin at a cut-off of greater than 22.23 μmol/L had a sensitivity of 0.84 (95 % CI 0.65 to 0.94) and a specificity of 0.91 (0.86 to 0.94). Bilirubin at a cut-off of greater than twice the normal limit, had a sensitivity of 0.42 (95 % CI 0.22 to 0.63) and a specificity of 0.97 (95 % CI 0.95 to 0.99). For ALP at a cut-off of greater than 125 IU/L, sensitivity was 0. 92 (95 % CI 0.74 to 0.99) and specificity was 0.79 (95 % CI 0.74 to 0.84). For ALP at a cut-off of greater than twice the normal limit, sensitivity was 0.38 (95 % CI 0.19 to 0.59) and specificity was 0.97 (95 % CI 0.95 to 0.99) [131, 132].

#### Statement 7.2 At AUS, the visualization of CBDS is a very strong predictor of choledocholithiasis. (LoE 5 GoR D). Indirect signs of stone presence such as increased diameter of common bile duct are not sufficient to identify ACC patients with choledocholithiasis and further diagnostic tests are needed. (LoE 1 GoR A)

AUS is the preferred imaging technique to diagnose ACC. Simultaneously, the common bile duct can be visualized and investigated. A recently published meta-analysis investigated the diagnostic potential of ultrasound [131]: sensitivity ranged from 0.32 to 1.00 with a summary sensitivity of 0.73 (95 % CI 0.44 to 0.90), and specificity ranged from 0.77 to 0.97 with a summary specificity of 0.91 (95 % CI 0.84 to 0.95). In a retrospective analysis, Boys et al [133] demonstrated that AUS mean common bile duct diameter in ACC patients without and with CBDS was 5.8 and 7.1 mm, respectively (*P* value = 0.004). Diameter >10 mm was associated with 39 % incidence of CBDS, while diameter < 9.9 mm was associated with common bile duct stones in 14 %. The authors’ conclusion was that AUS common bile duct diameter is not sufficient to identify patients at significant risk for CBDS.

#### Statement 7.3 Liver biochemical tests, including ALT, AST bilirubin, ALP, gamma glutamyl transferase (GGT), AUS should be performed in all patients with ACC to assess the risk for CBS. (LoE 2 GoR B)

Several predictive scores of CBDS have been proposed and validated but none are specific for ACC. The implementation of these predictive scores in clinical practice is poor [126, 134–138]. All combine the same clinical variables differently. Hugrier et al combined diameter of common bile duct > 12 mm, gallstones < 10 mm, advanced age and symptomatic disease; Barkun et al combined age > 55, elevated serum bilirubin, dilated common bile duct and evidence of CBDS; Menezes combined age > 55, male sex, ascending cholangitis, dilated common bile duct, CBDS, and abnormal liver tests; Soltan et al included history of symptomatic disease, abnormal liver tests, dilated common bile duct and presence of CBDS; Sun et al included male sex, abnormal liver test and dilated common bile duct; Sarli et al combined positive AUS and abnormal liver tests. The American Society of Gastrointestinal Endoscopy and the Society of American of Gastrointestinal Endoscopic Surgeons combined the various published validated clinical scores and proposed a risk stratification of CBDS in three different classes: low risk (<10 %), moderate (10 to 50 %) and high risk (> 50 %), based on the presence of predictive factors for having CBDS in its guidelines [139]. This proposed classification has clear clinical implications. Patients with a low risk of CBDS should be operated upon without further investigation. Patients with moderate risk should be interrogated with a second level examination: preoperatively by endoscopic ultrasound (EUS) or magnetic resonance cholangiopancreatography (MRCP) or intraoperatively by laparoscopic ultrasound or laparoscopic cholangiography, to select patients who need stone removal prior, during or after surgery. Patients with high risk of CBDS should undergo directly preoperative diagnostic and therapeutic ERCP.

#### Statement 7.4 common bile duct stone risk should be stratified according to the proposed classification, modified from the American Society of Gastrointestinal Endoscopy and the Society of American Gastrointestinal Endoscopic Surgeon Guidelines (LoE 5 GoR D)

ASGE guidelines seem to be the best tool available for the diagnosis and the management of CBDS during ACC [139]. However, according to this classification high risk patients have a probability of having CBDS > 50 %: this means that up to 49 % of patients that undergo ERCP may have no CBDS and, given the potential complications of ERCP, this is not acceptable. For this reason we prefer a more cautious approach: only patients with evidence of CBDS at AUS should be considered at high risk of CBDS and should undergo directly diagnostic and therapeutic ERCP; patients with total serum bilirubin > 4 mg/dL, or enlarged common bile duct diameter at AUS plus bilirubin level 1.8 to 4 mg/dL should be considered as moderate risk and should undergo second level investigation such as EUS/MRCP, or intraoperative Laparoscopic ultrasound/cholangiography to avoid the ERCP complications. See Table 4 for the modified risk stratification.

**Table 4:** Predictive factors and risk classes for choledocholithiasis

Predictive factor for choledocholithiasis	
Very strong	Evidence of common bile duct stone at abdominal ultrasound
Strong	Common Bile duct diameter > 6 mm (with gallbladder in situ)
	Total Serum Bilirubin > 4 mg/dL
	Bilirubin level 1.8 to 4 mg/dL
Moderate	Abnormal liver biochemical test other than bilirubin
	Age older than 55 years
	Clinical gallstone pancreatitis
Risk class for choledocholithiasis	
High	Presence of any VERY STRONG
Low	No predictors present
Intermediate	All other patients

Modified from [139]

#### Statement 7.5 Patients with moderate risk for choledocholithiasis should undergo preoperative MRCP, EUS, intraoperative cholangiography, or Laparoscopic ultrasound depending on the local expertise and availability. (LoE 1 GoR A)

Two preoperative imaging techniques are available for the detection of CBDS, MRCP and EUS. These diagnostic tools, according to the ASGE guidelines [139] should be reserved for patients with moderate risk for choledocholithiasis and have been shown to delay definitive ACC treatment [133]. On the other hand, these tests could exclude the presence of CBDS with high diagnostic accuracy, thereby avoiding further invasive procedures such ERCP or intraoperative cholangiography and their complications. In fact, the implementation of these techniques resulted in a reduction of ERCP ranging from 30 to 75 % in non-selected patients. [140–142]. A Cochrane meta-analysis compared these two different techniques [143]: both had good diagnostic accuracy and did not differ significantly with a summary sensitivity of 95 % for EUS and 93 % for MRCP and a summary specificity of 97 and 96 % respectively. As noted by some authors interpreting similar results, considerations other than diagnostic efficacy (local availability, costs, expertise, delay of surgery) might be important when deciding which imaging method to use [144].

#### Statement 7.6 Patients with high risk for choledocholithiasis should undergo preoperative ERCP, intraoperative cholangiography, Laparoscopic ultrasound, depending on the local expertise and the availability of the technique. (LoE 1 GoR A)

ERCP has both a diagnostic and therapeutic role in the management of choledocholithiasis but is an invasive procedure with potential severe complications. The literature emphasizes that diagnostic ERCP has risks. Morbidity associated with diagnostic ERCP includes pancreatitis, cholangitis, haemorrhage, duodenal perforation, or allergy to contrast. These occur in 1 to 2 % and increase to 10 % when associated with sphincterotomy [145–148]. On the other hand intraoperative cholangiography significantly increases the length of surgery [149] and requires dedicated staff in the operating room. This is not always available, especially in the acute setting with non-planned operation as in ACC. Positive findings on intraoperative cholangiography lead to intraoperative management of CBDS with additional operative time. A recently published meta-analysis compared the two techniques [131]: for ERCP, the summary sensitivity was 0.83 (95 % confidence interval 0.72 to 0.90) and specificity was 0.99 (95 % CI 0.94 to 1.00). For intraoperative cholangiography, the summary sensitivity was 0.99 (95 % CI 0.83 to 1.00) and specificity was 0.99 (95 % CI 0.95 to 1.00). Sensitivities showed a weak statistical difference (*p* = 0.05) but due to the quality and the methodology of the included studies, the two diagnostic techniques should be considered equivalent. Recently, Laparoscopic ultrasound has been introduced for the detection of CBDS. A recent meta-analysis has shown that intraoperative cholangiography and Laparoscopic ultrasound have the same pooled sensitivity and similar pooled specificity for the detection of CBDS [150]. As in the case of intraoperative cholangiography, intraoperative evidence of CBDS leads to intraoperative management of common bile duct with additional operating time.

#### Statement 7.7 CBDS could be removed preoperatively, intraoperatively, or postoperatively according to the local expertise and the availability of the technique. (LoE 1 GoR A)

CBDS could be removed with varying techniques in different timings: preoperative ERCP with sphincterotomy, intraoperative ERCP with sphincterotomy, laparoscopic or open common bile duct exploration, or post-operative ERCP with sphincterotomy. A systematic review assessed the difference between these different techniques [151]. No differences in terms of morbidity, mortality and success rate were reported comparing these methods. Therefore, these techniques should be considered suitable options. Another meta-analysis investigated two different techniques for ERCP plus sphincterotomy: preoperative or intraoperative with the rendezvous technique [152]. These two techniques were equal in safety and efficacy; intraoperative technique reduced the risk for post-ERCP pancreatitis, but obviously requires dedicated staff in the theatre and prolongs the length of surgery.

### Alternative treatments for high risk patients

#### Statement 8.1 Gallbladder drainage, together with antibiotics, converts a septic cholecystitis into a non-septic condition; however the level of evidence is poor (LoE 4, GoR C)

As already stated, the definitive treatment of ACC is early laparoscopic cholecystectomy. However some patients may not be suitable candidates for surgery, due to co-morbidities. Cholecystectomy for ACC in the elderly and in high risk patients has always been considered a high-risk procedure with a reported morality up to 19 % [153]. Recently published articles show that emergency cholecystectomy for ACC could be considered a feasible and safe procedure [89, 153–157].

Gallbladder drainage, also known as percutaneous cholecystostomy (PC) is a potential alternative to cholecystectomy in high-risk patients, but its role is difficult to determine because different definitions are used to identify “high-risk” patients. Gallbladder drainage decompresses the infected bile or pus in the gallbladder, removing the infected collection without removing the gallbladder. The removal of the infected material, in addition to antimicrobial therapy, can result in a reduced inflammation with an improvement of the clinical condition. Several case series, retrospective and observational studies exist on cholecystostomy. A systematic review of the literature included 53 studies with 1918 patients outlining a high success rate of the procedure (85.6 %) with a low procedure related mortality (0.36 %); however, the 30-day mortality was 15.4 % [153]. A major limitation of the study was the inclusion of patients with both acute acalcolus cholecystitis and ACC. After the aforementioned review, about 27 further observational studies have been published, confirming that the groups considered in the studies, their inclusion criteria, the results and even the conclusions reached by different authors are largely non-homogeneous [158]. With these limitations in mind, the reported in-hospital mortality for cholecystostomy varies between 4 and 50 % and morbidity ranges between 8.2 and 62 %.

#### Statement 8.2 Among standardized gallbladder drainage techniques percutaneous transhepatic gallbladder drainage (PTGBD) is generally recognized as the preferred technique due to the ease and the reduced costs. (LoE 4, GoR C)

Cholecystostomy can be performed with several different techniques as summarized well by the TG [159]. These include PTGBD, percutaneous transhepatic gallbladder aspiration (PTGBA), endoscopic naso-biliary gallbladder drainage, endoscopic gallbladder stenting, and EUS-guided gallbladder drainage via the antrum of the stomach and the duodenum. A controlled trial by Ito et al. [160] compared PTGBD with PTGBA. All patients with ACC were treated conservatively and patients who showed no improvements after 24 h were randomized to receive either PTGBD or PTGDA. PTGBD was superior to gallbladder aspiration in terms of clinical effectiveness with the same complication rate as gallbladder aspiration. However this trial included high risk and low risk patients. No other good quality evidence exists on which is the best gallbladder drainage technique. Finally, in case of evidence of cystic duct obstruction, PTGDB should be, even more, the preferred technique for gallbladder drainage.

#### Statement 8.3 PC could be considered as a possible alternative to surgery after the failure of conservative treatment in a small subset of patients unfit for emergency surgery due to their severe co-morbidities (LoE 2 GoR B)

TG on ACC [11] consider the gallbladder drainage as mandatory in the severe grade (according to the Tokyo classification [12]) acute cholecystitis and also suggest its use in the moderate grade if conservative treatment fails. The panel of the Tokyo Guidelines states that it is known to be an effective option in critically ill patients, especially in elderly patients and patients with complications; however, there is a lack of good quality evidence to support the statement. Hatzidakis et al. published in 2002 a randomized trial comparing PC with conservative treatment in patients with acute acalcolus cholecystitis or ACC [161]: there were no significant differences in mortality and morbidity. Akyurek et al published in 2005 a trial where patients with ACC were randomized to receive PC followed by early laparoscopic cholecystectomy or conservative treatment followed by delayed laparoscopic cholecystectomy [162]. There were no differences in term of mortality and morbidity; PC plus early laparoscopic cholecystectomy resulted in a reduction of the length of stay and of costs. Melloul et al. in 2011 published a retrospective case control study in critically ill patients with biliary sepsis treated by early laparoscopic cholecystectomy or PC [163]: mortality was not different between the two treatments but early laparoscopic cholecystectomy was associated with significantly higher complication rate. A Spanish retrospective study [164] compared critically ill patients with ACC who underwent PC or early laparoscopic cholecystectomy. They found a significantly higher mortality rate in the PC group; however this study is of poor quality and has several limitations such as the retrospective study design and the selection bias. A Cochrane systematic review by Gurusamy et al. investigated the role of cholecystostomy: authors included the only two randomized trials, both at high risk of bias, concluding that “we are unable to determine the role of percutaneous cholecystostomy in the clinical management of high-risk surgical patients with acute cholecystitis” [165]. Currently, the CHOCOLATE trial is ongoing [161]: it is a randomized controlled trial comparing PC with early laparoscopic cholecystectomy in critically ill patients (APACHE score 7–14) with ACC; results may clarify the real role of the percutaneous drainage. Gallbladder drainage has been even described as a procedure reserved for those patients who failed the conservative treatment after a variable time of 24 to 48 h. A prospective study by Barak et al. [166] reported age above 70 years, diabetes, tachycardia, and a distended gallbladder at admission as predictors for the failure of conservative treatment at 24 h follow-up, while WBC > 15,000 cell/mm3, elevated temperature, and age above 70 years were predictors for the failure of conservative treatment at 48 h follow-up. There is no specific antibiotic regimen to be prescribed alongside PC. None of the examined studies reported the specific drug agent. No evidence exists supporting the need for a peculiar antibiotic regimen. For the antimicrobial therapy, please see the dedicated section. At the present time, PC seems to be a safe and effective procedure in critically ill patients with ACC. However, no evidence supports its superiority toward the conservative treatment or early laparoscopic cholecystectomy.

#### Statement 8.4 delayed laparoscopic cholecystectomy could be offered to patients after reduction of operative and anesthesiology- related risks to reduce further hospitalization (LoE 5 GoR D)

De Mestral et al. published a large retrospective epidemiological analysis in 2012 showing that only 40 % of patient underwent delayed laparoscopic cholecystectomy after PC; the 1 year readmission rate for patients who did not undergo delayed laparoscopic cholecystectomy after PC was 49 % with an in-hospital mortality of 1 % [10]. No randomized trial comparing the need for delayed laparoscopic cholecystectomy exists currently.

## Conclusion: grey areas and opportunities for future research

After achieving the consensus for all the statements, the participants to the Consensus Conference voted for the WSES algorithm on ACC which is reported in Fig. 1.

Based on the evidence included in the present guidelines, it can be stated that early laparoscopic cholecystectomy is the best therapeutic approach for ACC and that post-operative antibiotics are not necessary in cases of uncomplicated cholecystitis. Moreover, studies providing a high level of evidence on the management of associated CBDS have also been published. Visualisation of CBDS by AUS is a good predictor; patients with a high risk of CBDS should have a pre-operative ERCP; patients with a moderate risk should have non-invasive pre-operative investigation. However in both cases intra-operative exploration according to the local expertise has been reported as a recommended option with a high level of evidence. Furthermore we observed lack of studies investigating the cost savings of transcystic duct common bile duct removal of small stones.

The recommendations on the surgical treatment of ACC are however limited to patients who may be good candidates for urgent surgery. Grey areas still remain in the cases of patients not fit for urgent surgery or for laparoscopic surgery secondary to general conditions.

Diagnosis may be assessed by clinical, laboratory data and AUS but with such a diagnostic approach results appear controversial and supported by a limited number of high quality studies. A radiological investigation such as HIDA may be required to reach a diagnostic certainty. Since symptomatic gallbladder stones are, in any case, an indication for laparoscopic cholecystectomy, the former diagnostic uncertainty may not be relevant in healthy patients and the latter invasive radiological investigation should therefore be applied only in high-risk patients.

There is however no consensus on the evaluation of the operative risk. These WSES guidelines define the patient condition in lieu of the cholecystitis severity score as underlined in the TG13. This approach could favour a tailored therapy on patient’s condition. Although the role of percutaneous cholecystostomy after failed conservative treatment in those patients not fit for surgery secondary to severe co-morbidities has been reported, the present guidelines have failed to find valuable criteria for the definition of such high-risk patients. Data on criteria for a definition of a high-risk patient other than that of septic shock, are scarce and of poor level of evidence. This is an area for research to improve the management of patients with ACC.

According to some high quality studies, subtotal cholecystectomy and low threshold for conversion should be recommended in cases of severe acute inflammation of the gallbladder at operation. Although the threshold for conversion strongly depends on the experience and skills of the surgeon, we support the development of an intraoperative score to help the surgeon in the decision to complete the operation by partial cholecystectomy and/or by open approach when “the critical view of safety” cannot be reached without adding risk.

## Abbreviations

ACC, acute calculous cholecystitis; APACHE II, acute physiology and chronic health evaluation II; ASA, American Society of Anaesthesiology; AUS, abdominal ultrasound 37; CBD, common bile duct; CBDS, common bile duct stones 37; DLC, delayed laparoscopic cholecystectomy; ELC, early laparoscopic cholecystectomy; ERCP, endoscopic retrograde cholangiopancreateography; EUS, endoscopic ultrasound; GoR, grade of recommendation; IOC, intraoperative cholangiography; LC, laparoscopic cholecystectomy; LoE, level of evidence; LUS, laparoscopic ultrasound; MRCP, magnetic resonance cholangiopancreateography; OC, Organization Committee; Ppossum, portsmouth physiological and operative severity score for the enUmeration of mortality and morbidity; SC, Scientific Committee; SS, Scientific Secretariat; TG, Tokyo guidelines; WSES, World Society of Emergency Surgery

## Figures and Tables

**Fig. 2:**
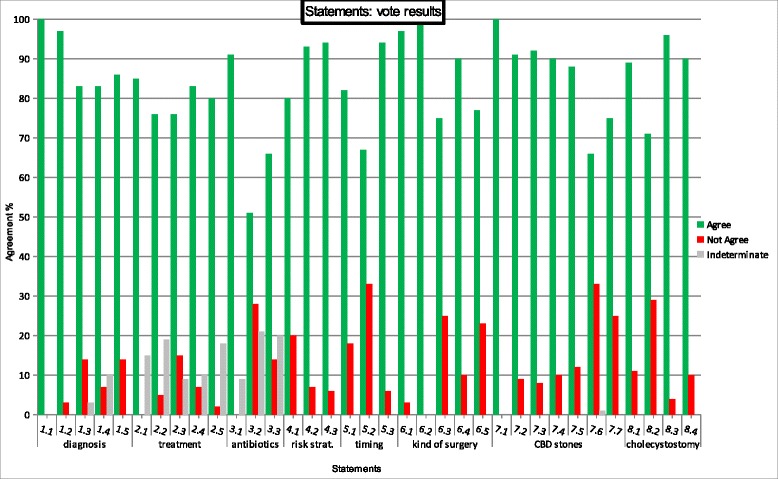
Vote results of statements

## Appendix 2

**Table 5:** WSES Guidelines statements

Topic	#	LoE	GoR	
Diagnosis	1.1	4	C	There is no single clinical or laboratory finding with sufficient diagnostic accuracy to establish or exclude acute cholecystitis. Combination of detailed history, complete clinical examination, and laboratory tests may strongly support the diagnosis of ACC
	1.2	2	B	Abdominal ultrasound (AUS) is the preferred initial imaging technique for patients who are clinically suspected to have ACC because of its lower cost, better availability, lack of invasiveness, and high accuracy for gallbladder stones.
	1.3	3	C	exploration is a fairly reliable investigation method but its sensitivity and specificity for diagnosing ACC may be relatively low according to the adopted AUS criteria.
	1.4	2	B	Evidence on the diagnostic accuracy of computed tomogram (CT) is scarce. While diagnostic accuracy of magnetic resonance imaging (MRI) might be comparable to that of AUS, insufficient data are available to support this. Hepatobiliary iminodiacetic acid scan (HIDA scan) has the highest sensitivity and specificity for AC, although its scarce availability, long time required to perform the test, and exposure to ionizing radiation limit its use.
	1.5	4	C	Combining clinical, laboratory and imaging investigations is recommended, although the best combination is not yet known.
Treatment	2.1	2	B	There is no role for gallstones dissolution, drugs or extra-corporeal shock wave lithotripsy (ESWL) or a combination in the setting of ACC.
	2.2	4	C	Since there are no reports on surgical gallstone removal in the setting of ACC, surgery in the form of cholecystectomy remains the main option
	2.3	3	C	Surgery is superior to observation of ACC in the clinical outcome and shows some cost-effectiveness advantages due to the gallstone-related complications and to the high rate of readmission and surgery in the observation group
	2.4	2	C	Antibiotics should be suggested as supportive care; they are effective in treating the first episode of ACC but a high rate of relapse can be expected. Surgery is more effective than antibiotics alone in the treatment of ACC.
	2.5	3	C	Cholecystectomy is the gold standard for treatment of ACC.
	2.6	5	D	If surgery is not available, medications such as antibiotics and analgesic should be prescribed and the patients should be referred to a surgical center (depending upon the general condition) due to the high rate of gallstone-related events.
Antibiotics	3.1	1	B	Patients with uncomplicated cholecystitis can be treated without post-operative antibiotics when the focus of infection is controlled by cholecystectomy
	3.2	3	B	In complicated cholecystitis, the antimicrobial regimens depend on presumed pathogens involved and risk factors for major resistance patterns
	3.3	3	C	The results of microbiological analysis are helpful in designing targeted therapeutic strategies for individual patients to customize antibiotic treatment and ensure adequate antimicrobial coverage in patients with complicated cholecystitis and at high risk for antimicrobial resistance.
High risk patients	4.1	3	B	Patient’s age above 80 in ACC is a risk factor for worse clinical behaviour, morbidity and mortality.
	4.2	3	C	The co-existence of diabetes mellitus does not contraindicate urgent surgery but must be re-considered as a part of the overall patient comorbidity.
	4.3	4	C	Currently, there is no evidence of any scores in identifying patient’s risk in surgery for ACC. ASA, POSSUM and APACHE II are correlated to surgical risk in patients with gallbladder perforation, higher accuracy being for APACHE II. However, APACHE II is built to predict morbidity and mortality in the patients admitted to ICU: its use as a preoperative score should be considered as an extension usage from the original concept. Therefore, prospective and multicentre studies to compare different risk factors and scores are necessary
Timing	5.1	1	A	ELC is preferable to DLC in patients with ACC as long as it is completed within 10 days of onset of symptoms.
	5.2	2	B	ELC should not be offered for patients beyond 10 days from the onset of symptoms unless symptoms suggestive of worsening peritonitis or sepsis warrant an emergency surgical intervention. In people with more than 10 days of symptoms, delaying cholecystectomy for 45 days is better than immediate surgery.
	5.3	1	A	ELC should be performed as soon as possible but can be performed up to 10 days of onset of symptoms. However, it should be noted that earlier surgery is associated with shorter hospital stay and fewer complications.
Type of surgery	6.1	2	B	In ACC, a laparoscopic approach should initially be attempted except in case of absolute anaesthesiology contraindications or septic shock.
	6.2	1	A	LC for ACC is safe, feasible, with a low complication rate and associated with shortened hospital stay.
	6.3	3	C	Among high-risk patients, in those with Child A and B cirrhosis, advanced age >80, or pregnant women, laparoscopic cholecystectomy for ACC is feasible and safe.
	6.4	3	A	Laparoscopic or open subtotal cholecystectomy is a valid option for advanced inflammation, gangrenous gallbladder, or any setting of the “difficult gallbladder” where anatomy is difficult to recognize and main bile duct injuries are moe likely.
	6.5	3	B	In case of local severe inflammation, adhesions, bleeding in Calot’s triangle or suspected bile duct injury, conversion to open surgery should be strongly considered.
Associated common bile duct stones	7.1	2	B	Elevation of liver biochemical enzymes and/or bilirubin levels are not sufficient to identify ACC patients with choledocholithiasis and further diagnostic tests are needed.
	7.2	1	A	At AUS, the visualization of CBDS is a very strong predictor of choledocholithiasis. Indirect signs of stone presence such as increased diameter of CBD are not sufficient to identify ACC patients with choledocholithiasis and further diagnostic tests are needed.
	7.3	2	B	Liver biochemical tests, including ALT, AST bilirubin, ALP, gamma glutamyl transferase (GGT), AUS should be performed in all patients with ACC to assess the risk for CBS.
	7.4	5	D	CBD stone risk should be stratified according to the proposed classification, modified from the American Society of Gastrointestinal Endoscopy and the Society American of Gastrointestinal Endoscopic Surgeon Guidelines.
	7.5	1	A	Patients with moderate risk for choledocholithiasis should undergo preoperative MRCP, EUS, intraoperative cholangiography (IOC), or LUS depending on the local expertise and availability.
	7.6	1	A	with high risk for choledocholithiasis should undergo preoperative ERCP, IOC, LUS, depending on the local expertise and the availability of the technique.
	7.7	1	A	CBDS could be removed preoperatively, intraoperatively, or postoperatively according to the local expertise and the availability of the technique.
Alternative treatments	8.1	4		Gallbladder drainage, together with antibiotics, converts a septic cholecystitis into a non-septic condition; however the level of evidence is poor.
	8.2	4	C	Among standardized gallbladder drainage techniques percutaneous transhepatic gallbladder drainage (PTGBD) is generally recognized as the preferred technique due to the ease and the reduced costs.
	8.3	2	B	PC could be considered as a possible alternative to surgery after the failure of conservative treatment in a small subset of patients unfit for emergency surgery due to their severe co-morbidities.
	8.4	5	D	DLC could be offered to patients after reduction of operative and anesthesiology- related risks to reduce further hospitalization.
