# Erratum to: 2016 WSES guidelines on acute calculous cholecystitis

**DOI:** 10.1186/s13017-016-0088-z

**Published:** 2016-11-04

**Authors:** L. Ansaloni, M. Pisano, F. Coccolini, A. B. Peitzmann, A. Fingerhut, F. Catena, F. Agresta, A. Allegri, I. Bailey, Z. J. Balogh, C. Bendinelli, W. Biffl, L. Bonavina, G. Borzellino, F. Brunetti, C. C. Burlew, G. Camapanelli, F. C. Campanile, M. Ceresoli, O. Chiara, I. Civil, R. Coimbra, M. De Moya, S. Di Saverio, G. P. Fraga, S. Gupta, J. Kashuk, M. D. Kelly, V. Khokha, H. Jeekel, R. Latifi, A. Leppaniemi, R. V. Maier, I. Marzi, F. Moore, D. Piazzalunga, B. Sakakushev, M. Sartelli, T. Scalea, P. F. Stahel, K. Taviloglu, G. Tugnoli, S. Uraneus, G. C. Velmahos, I. Wani, D. G. Weber, P. Viale, M. Sugrue, R. Ivatury, Y. Kluger, K. S. Gurusamy, E. E. Moore

**Affiliations:** 1 General Surgery I, Papa Giovanni XXIII Hospital, Piazza OMS 1, Bergamo, 24127 Italy; 2 Department of Surgery, UPMC, University of Pittsburgh School of Medicine, Pittsburgh, PA USA; 3 Department of Surgical Research, Medical Univeristy of Graz, Graz, Austria; 4 Department of Emergency and Trauma Surgery of the University Hospital of Parma, Parma, Italy; 5 Department of General Surgery, Adria Civil Hospital, Adria, RO Italy; 6 University Hospital Southampton, Southampton, UK; 7 Department of Traumatology, John Hunter Hospital and University of Newcastle, Newcastle, NSW Australia; 8 Acute Care Surgery, Queen’s Medical Center, School of Medicine of the University of Hawaii, Honolulu, HI USA; 9 Department of Surgery, IRCCS Policlinico San Donato, University of Milan Medical School, Milan, Italy; 10 University of Verona, Verona, Italy; 11 Unit of Digestive, Hepato-Pancreato-Biliary Surgery and Liver Transplantation, Henri Mondor Hospital AP-HP, Université Paris Est-UPEC, Créteil, France; 12 Surgical Intensive Care Unit, Department of Surgery, Denver Health Medical Center, University of Colorado School of Medicine, Denver, USA; 13 General Surgery - Day Surgery Istituto Clinico Sant’Ambrogio, Insubria University, Milan, Italy; 14 Ospedale San Giovanni Decollato - Andosilla, Civita Castellana, Italy; 15 Emergency Department, Trauma Center, Niguarda Hospital, Milan, Italy; 16 Department of Surgery, Auckland City Hospital, Auckland, New Zealand; 17 Division of Trauma, Surgical Critical Care, Burns, and Acute Care Surgery, University of California San Diego Health Sciences, San Diego, CA USA; 18 Harvard University, Cambridge, MA USA; 19 General, Emergency and Trauma Surgery, Maggiore Hospital Trauma Center, Bologna, Italy; 20 Division of Trauma Surgery, University of Campinas, Campinas, SP Brazil; 21 Department of Surgery, Government Medical College, Chandigarh, India; 22 Tel Aviv University Sackler School of Medicine, Assia Medical Group, Tel Aviv, Israel; 23 Acute Surgical Unit, Canberra Hospital, Canberra, ACT Australia; 24 Surgical Department, Mozyr City Hospital, Mozyr, Belarus; 25 Erasmus MC Rotterdam, Rotterdam, Holland Netherlands; 26 University of Arizona, Tucson, AZ USA; 27 Meilahti Hospital, Helsinki, Finland; 28 Department of Surgery, Harborview Medical Center, Seattle, WA USA; 29 Department of Trauma, Hand, and Reconstructive Surgery, University Hospital, Goethe-University Frankfurt, Frankfurt, Germany; 30 Department of Surgery, University of Florida, Gainesville, FL USA; 31 First General Surgery Clinic, University Hospital St. George/Medical University, Plovdiv, Bulgaria; 32 Department of Surgery, Macerata Hospital, Macerata, Italy; 33 Shock Trauma Center, Critical Care Services, University of Maryland School of Medicine, Baltimore, MD USA; 34 Denver Health Medical Center, Denver, CO USA; 35 Taviloglu Proctology Center, Istanbul, Turkey; 36 Department of Surgery, Medical University of Graz, Graz, Austria; 37 Emergency Surgery, and Surgical Critical Care, Massachusetts General Hospital, Boston, MA USA; 38 DHS, Srinagar, Kashmir India; 39 Trauma and General Surgery & The University of Western Australia, Royal Perth Hospital, Perth, Australia; 40 Infectious Disease Unit, Teaching Hospital, S. Orsola-Malpighi Alma Mater Studiorum, University of Bologna, Bologna, Italy; 41 Letterkenny University Hospital & Donegal Clinical Research Academy, Donegal, Ireland; 42 Virginia Commonwealth University, Richmond, VA USA; 43 Division of General Surgery, Rambam Health Care Campus, Haifa, Israel; 44 Royal Free Campus, University College London, London, UK

## Erratum

Unfortunately, the original version of this article [1] contained an error in the spelling of an author’s name. The surname is written as Koka, but should be spelt as Khokha.
